# Enhancement of the Bond Strength and Reduction of Wafer Edge Voids in Hybrid Bonding

**DOI:** 10.3390/mi13040537

**Published:** 2022-03-29

**Authors:** Yeoun-Soo Kim, Thanh Hai Nguyen, Sung-Hoon Choa

**Affiliations:** 1 Graduate School of Nano IT Design Fusion, Seoul National University of Science and Technology, Seoul 01811, Korea; kimys95@seoultech.ac.kr; 2 Institute of Science and Technology, Ministry of Public Security, Hanoi 11355, Vietnam; nguyethanhhai266@gmail.com

**Keywords:** hybrid bonding, bond strength, edge void, plasma conditions, wafer warpage

## Abstract

The hybrid wafer bonding technique is drawing much interest in relation to three-dimensional integration technology, and its areas of application are expanding from image sensors to semiconductor memory packages. In hybrid bonding, the bond strength and void formation are the main issues influencing the performance, reliability, and yield of the bonding. In this study, we systematically investigate several parameters that affect both the bond strength and void formation, including the plasma gas, plasma power, and surface roughness. In particular, the effects of the wafer warpage on void formation were investigated. As O_2_ gas was used during plasma activation, the highest oxide growth rate and strongest bond strength were achieved. The bond strength improved when the oxide thickness was increased. An increase in the low-frequency plasma power improved the bond strength. However, when the plasma power increased further, the surface roughness increased due to the ion bombardment effect during the use of the plasma, resulting in a reduction in the bond strength. Therefore, optimization of the plasma power is required to improve the bond strength. It was found that the wafer warpage was also an important parameter which affected the formation of edge voids. The wafers with residual compressive stress exhibited fewer edge voids than those with tensile stress. Several methods to minimize edge void formation in wafers are proposed. The present study will provide practical guidelines to enhance the quality and yield of the bonding process and devices.

## 1. Introduction

The demand for high speeds and high-performance levels of image sensors continues to increase in diverse applications, such as autonomous vehicles, security, robotics, the IoT (internet of things), and artificial intelligence technologies [1,2,3]. The three-dimensional (3D) stacked image sensor using 3D integration technology has emerged as a new key technology to meet these demands [4]. Three-dimensional integration technology provides an integrated system in which cells and logic devices are vertically stacked and connected. Recently, the back side illumination (BSI) image sensor started to utilize the 3D integration process to overcome the limitations posed by the shrinkage of the pixel size [5,6,7]. The key technologies of 3D integration are the wafer-to-wafer bonding and the ultra-wafer thinning processes. Three-dimensional integration technologies can be classified into the through silicon via (TSV) method and the metal interconnection method depending on the electrical connection method of the cell and logic [8]. Hybrid bonding refers to a direct bond that combines a dielectric silicon oxide bond and a metal bond of a sensor and a logic wafer without a TSV to form electrical interconnections such as copper (Cu)/dielectric layer hybrid bonding [9]. Recently, hybrid bonding technology has expanded from image sensors to the semiconductor packaging technologies of NAND flash memory and DRAM products.

Much of the work has focused on investigations of the physical mechanisms [10] and fabrication optimization [11] of hybrid bonding technology. However, hybrid bonding technology is still associated with many challenging issues related to yields and reliability. Examples include the bond strength, void generation, misalignment, and contamination. The bonding quality is influenced by many factors, such as the cleanliness and roughness of the surface [12], surface activation [13], the annealing conditions used [14], and copper recession and protrusion [15]. During the fabrication of semiconductors, there is a need to improve the bond strength at low temperatures in order to prevent melting of the metal layer and changes in device characteristics during the bonding process. Therefore, plasma pretreatment and hydrophilic bonding techniques were introduced to improve adhesion at a low annealing temperature [16,17].

The warpage of the wafer is also crucial for a high yield and reliability of hybrid bonding, particularly when the number of stacked wafers increases [18]. It was known that deformed bonded wafers caused by differences in the thermal expansion of the neighboring materials (or residual stress) will affect the misalignment. However, there have been few studies on the effect of wafer warpage on void formation and yield of the bonding.

In this study, we focus on the bond strength and formation of edge voids, which are the main factors impacting the yield and reliability of the hybrid bonding process. We systematically investigate the effects of different plasma activation conditions, plasma gases, and surface roughness levels on the bonding strength. In addition, given that it is known that edge voids can occur even in wafers with a very smooth surface roughness on the sub-nanometer level [19], for the first time we investigate the effects of wafer warpage on the formation of edge voids. Then, we proposed an efficient method to minimize the edge void formation.

## 2. Wafer Bonding Methods

**Figure 1:**
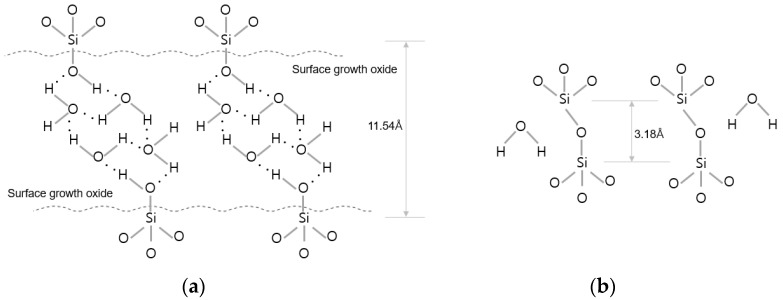
Description of binding mechanism during wafer bonding. (**a**) After the bonding process at room temperature. (**b**) After annealing process (≥150 °C).

There are various techniques in wafer bonding. The wafer bonding techniques commonly used in semiconductor manufacturing can be classified into the silicon direct bonding and hybrid bonding types (Cu-to-Cu and dielectric-to-dielectric bonding) depending on the bonding interface condition. Hybrid bonding refers to direct wafer bonding using Cu-to-Cu metal bonding combined with interlayer dielectric oxide bonding at the same time. Hybrid wafer bonding is generally conducted in the following process sequence: (1) the formation of dangling bonds and bonding between hydroxyl groups and water molecules through plasma activation using gases such as O_2_/N_2_/Ar; and (2) the removal of defects through deionized water cleaning and scrubbing. Then, the wafer surfaces are bonded together via van der Waals hydrogen bonds between two to three monolayers of water molecules and polar OH groups which terminate at both the native and thermal SiO_2_; and (3) the formation of van der Waals bonds between H_2_O molecules (Si-OH-(H_2_O)x-HO-Si) on the top and bottom wafer surfaces. Water molecules from the interface are removed, and covalent bonds are formed through annealing, as shown in Figure 1b [20].

Direct wafer bonding is generally conducted at room temperature and atmospheric pressure. However, the bond strength obtained under these conditions is less than 0.5 J/m^2^, which is low and insufficient for the subsequent wafer thinning process. An annealing process is necessary to increase the bond strength. However, the annealing temperature must be less than 400 °C to prevent the melting of the inter-metal layer and diffusion of the implanted dopant. Therefore, a hydrophilic bonding method that can secure sufficient bond strength at a relatively low temperature of 400 °C or less is commonly used. The goal of this method is to improve the bond strength by forming a Si-O-Si covalent bond due to the absorption of H_2_O molecules into the dielectric film or to release them to the outside during the annealing process [21]. The process from plasma activation to wafer bonding is performed in situ in the bonding machine.

Void formation during the bonding process is also the one of the critical issues affecting direct wafer bonding. Surface and morphological conditions such as the surface roughness, degree of cleanliness, and flatness are important factors to consider when attempting to control void formation and improve the bond strength. These factors can be influenced by certain aspects of the bonding pretreatment process, such as chemical mechanical polishing (CMP) and the plasma conditions during the bonding process. In particular, voids on the edges of the bonded wafer, known as edge voids, can form even under very clean surface conditions. The physical mechanism of edge void formation during the bonding process is explained by thermodynamics and fluid mechanics modeling between the wafers, known as the Joule–Thomson effect [22]. During the bonding process, the air between the wafer gap escapes from the center to the edge of the wafer. The air then expands at the surrounding atmospheric pressure due to the Joule–Thomson expansion effect. Upon the sudden pressure drop, the temperature of the air decreases slightly by a few degrees. The rapid cooling of the air leads to supersaturation of the water, resulting in nucleation of a water droplet at the edge of the wafer. Therefore, water droplet formation is the main source of edge voids during the bonding process [22]. In this study, in order to mitigate the formation of edge voids, we investigate the effects of wafer warpage caused by residual stress of the films and the bonding speed during the bonding process on the formation of edge voids.

## 3. Materials and Methods

### 3.1. Fabrication

**Figure 2:**
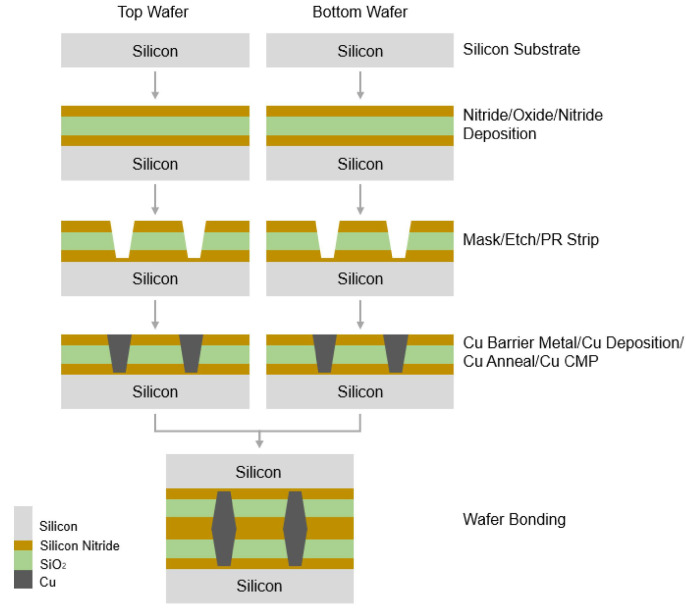
Schematic description of overall fabrication process for wafer bonding.

Hybrid bonding was conducted using a pair of 12 in (300 mm) silicon wafers. The thickness of each wafer was 775 μm. The bonding surface of the pair of wafers consists of a copper layer and a silicon nitride layer. The overall fabrication process for this work is shown as a schematic illustration in Figure 2. First, a multilayer silicon nitride/oxide/silicon nitride film was deposited onto the wafer by means of plasma-enhanced chemical vapor deposition (PECVD). Copper bonding pads for hybrid bonding were then formed through a process of masking, etching, and copper gap filling. Lastly, the CMP process was used to remove any excess copper and polish the wafer. The copper pads were patterned using i-line lithography with a pitch of 5 μm and width of 2 μm. The same structure and fabrication processes were applied to both the top and bottom wafer.

The surface roughness of the wafer is one of the critical factors determining the bond strength. Higher surface roughness will lead to a smaller real bonding contact area at the bonding interface, resulting in a reduction in the bond strength. Lower surface roughness will result in a larger real contact area and will make it easier to close the interfacial nanogap that forms during the post-annealing process [21], thereby increasing the bond strength. In this experiment, different levels of surface roughness were obtained using different sizes of abrasive particles in the slurry during the CMP process. The diameters of the abrasive particle range from 50 to 135 nm. The CMP process was conducted with a Reflexion^®^ LK CMP system (Applied Materials Co., Singapore). The urethane pad (DOW IC) was used in the CMP process. The copper oxide layer was removed, and then the Cu barrier metal layer was removed. Finally, 300 Å of the silicon nitride layer was removed. The different types of the slurries were used for each CMP process.

### 3.2. Wafer Bonding

The wafer bonding process involved plasma activation, deionized water (DIW) cleaning, and wafer bonding in sequence using EVG bonding equipment (GEMINIFB_XT, EV Group, Melaka, Malay Peninsula). During the bonding process, the dangling bonds and bonds between the hydroxyl groups and water molecules formed through plasma activation in a vacuum plasma chamber. The bonding process was conducted in the bonding module via the following sequence: (1) The top wafer was transported to the location of the bonding gap using a vacuum chuck. (2) The top vacuum chuck uses an inner and an outer vacuum state. During contact for wafer bonding, the vacuum state of the inner region of the top wafer was released, while the vacuum state of the outer region of the wafer was maintained. The center region of the top wafer was then brought into contact with the bottom wafer using a center pin. (3) During the contact, the vacuum state of the outer region was released simultaneously. After wafer bonding, the annealing of the bonded wafers was conducted at a temperature of 300 °C.

### 3.3. Plasma Activation

The plasma chamber had two driving electrodes, which were individually driven at two different frequencies in the kHz range by two separate power supplies. The bottom low-frequency component controlled the energy of the ions, and the top high-frequency component controlled the plasma density. The high frequency was 400 kHz, and the low frequency was 40 kHz. Plasma activation is an important factor that affects the bond strength through the formation of dangling bonds on the bonding surface. In addition, it plays an important role in changes of the surface roughness on the wafer, which affect the bonding speed and lead to edge void formation. Plasma activation involves complex chemical and physical reactions influenced by the plasma gas, the power, the frequency, and the vacuum state. Depending on the wafer surface conditions and film materials, various plasma gases were used to enhance the dangling bond formation and bond strength. In this experiment, the effects of different plasma gases, specifically N_2_, O_2_, and Ar, on the activation of the plasma were investigated. During the plasma activation process, the ion bombardment phenomenon affected the surface roughness. Therefore, we also investigated the effect of the low-frequency plasma power on the ion bombardment process. The plasma power at a low frequency was varied from 25 to 150 W, while the high-frequency plasma power was fixed at 60 W.

### 3.4. Characterization

The bond strength of the bonded wafer was evaluated by the double-cantilever-beam (DCB) method. During the DCB test, a razor blade was inserted at the edge locations on the bonded wafer and the wafer-to-wafer bond energy was obtained [23]. Observations of defects and edge voids at the bonding interface were conducted using a scanning acoustic microscope (SAM) (AW_PRO, Sonix, Zhubei City, Taiwan) with a 110 MHz transducer. Using this system, we could characterize voids larger than 200 mm in size. The surface roughness of the wafer was measured with an atomic force microscope (AFM). The measurement area for the AFM process was 20 μm × 20 μm on the surface of the silicon nitride layer. The center, middle, and edge regions on the wafer surface were each measured, and the average values of nine points were used. In this study, we investigated the effects of the warpage of the wafer on the generation of edge voids during the bonding process. The warpage of the wafer was controlled by varying the fabrication process of the silicon oxide and silicon nitride film. This warpage was measured with a warpage measurement system (128C2C, FSM Co., Denver, CO, USA) in the X and Y directions by two-line scans using a laser light source.

## 4. Results and Discussion

**Figure 3:**
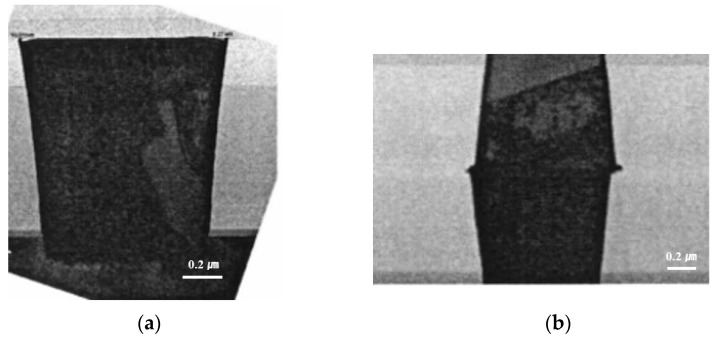
(**a**) The cross-sectional image of a fabricated wafer before bonding. (**b**) The cross-sectional image of the bonded wafer after completion of the annealing process including copper via and pad.

**Figure 4:**
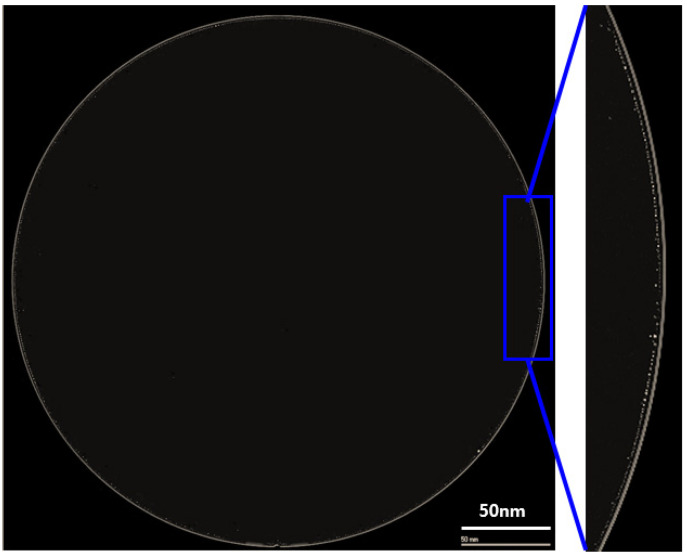
The top image of the bonded wafer using a c-SAM and edge void image shown in the yellow circles.

Figure 3a shows the cross-sectional transmission electron microscope (TEM) image of a fabricated wafer before bonding. It was found that the via hole was completely filled with copper without any defects or voids. Additionally, the copper dishing after the CMP process was very small, at less than 25 Å. Figure 3b shows the cross-sectional TEM image of the bonded wafer after completion of the annealing process. As shown in the figure, the wafer was well bonded without any misalignment or defects. In particular, the thickness of the copper oxide grown after annealing was around 50 Å. Therefore, the dishing effect can be negligible during the bonding process. Figure 4 exhibits a c-SAM image of a 12-inch bonded wafer after the annealing process. We found that the bonding was performed well. However, as shown in the figure, several voids were detected at the edge of the wafer (shown in the yellow circle).

The dangling bond formed in the vacuum plasma chamber has high energy and cannot be measured because it easily binds to oxygen when exposed to air. Therefore, the thickness of silicon oxide, which is a layer formed after the reaction, was monitored instead of the monitoring the dangling bond.

**Figure 5:**
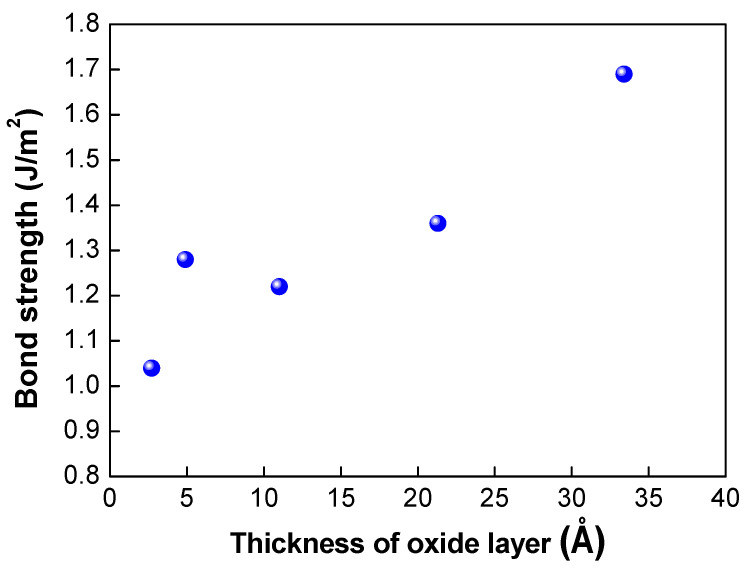
Correlation between thickness of the oxide layer grown on the nitride layer and bond strength.

Table 1 shows the results of the assessment of the bond strength of the bonded wafer and the growth rate of the oxide layer on the silicon nitride layer fabricated using different plasma activation gases (O_2_, N_2_, and Ar). The mixture gases of Ar/O_2_ and Ar/N_2_ were also used for plasma activation. In this experiment, the high frequency at the top electrode and low frequency at the bottom electrode were fixed at 400 and 40 kHz, respectively, and the top high power and bottom low power were correspondingly fixed at 60 W and 25 W. The growth of the oxide layer was measured by an ellipsometer before and after the plasma activation process. As shown in Table 1, the oxide growth rate indicates increases in the oxide thickness after plasma activation. When plasma activation was performed with O_2_ gas alone, the highest bond strength of 1.69 J/m^2^ and the highest oxide growth rate of 33.4 Å were achieved. In addition, plasma activation with Ar gas led to the lowest bond strength of 1.04 J/m^2^ and lowest oxide growth rate of 2.7 Å. The use of the mixture of Ar and O_2_ gases led to higher oxide growth of 21.3 Å and bond strength of 1.36 J/m^2^. The correlation between the bond strength and the oxide thickness is depicted in Figure 5. As the thickness of the oxide layer increased, the bond strength increased linearly, and the gas with a higher oxide growth rate showed higher bond strength, indicating that the thickness of the oxide layer is correlated with the dangling bond formation. As mentioned above, various gases can be used to enhance the plasma activation and dangling bond formation. Our results indicate that O_2_ gas is the most suitable gas for the silicon nitride film to enhance the bond strength.

Meanwhile, higher growth of copper oxide on the Cu bonding pad will have a somewhat negative effect on the bonding quality and bond strength during the bonding process. During the subsequent annealing process after bonding, the thickness of the copper oxide on the Cu bonding pad will also increase. However, the coefficient of thermal expansion (CTE) of the copper is greater than that of the brittle copper oxide (CuxOy). Therefore, during the annealing process, the Cu pad expands more than the copper oxide layer, resulting in the fracture of the copper oxide layer and forming a connection between the upper and lower Cu pads. In this regard, the growth of the silicon oxide is more important than the growth of the copper oxide. A higher growth rate of the silicon oxide will lead to higher bond strength.

During plasma activation, dangling bonds are formed on the surface of the silicon nitride layer, and polar hydroxyl groups become attached to the dangling bonds. The polar hydroxyl facilitates bonding with water molecules; therefore, wafer bonding is maintained through van der Waals bonding with water molecules. Plasma activation involves both a physical reaction and a chemical reaction. When O_2_ gas is used, a high oxide growth rate can be expected because relatively more chemical reactions favorable for oxide formation occur compared to when other gases are used. Numerous Si-O-Si covalent bonds were formed through the subsequent annealing process, and the bond strength was improved. Moreover, as the oxide growth rate increases, the dielectric oxide materials around the Cu pad will hold the Cu pad firmly during the bonding process, leading to higher bond strength.

**Figure 6:**
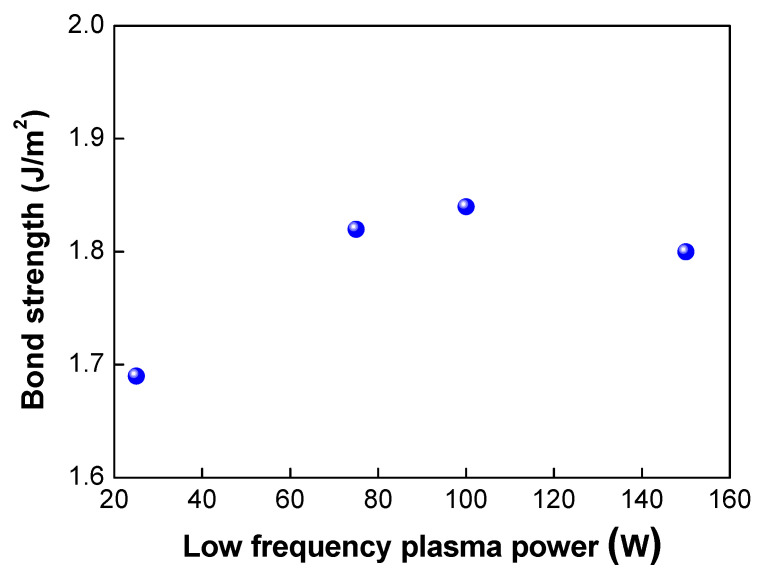
Changes in bonding strength when low frequency plasma power was increased.

**Figure 7:**
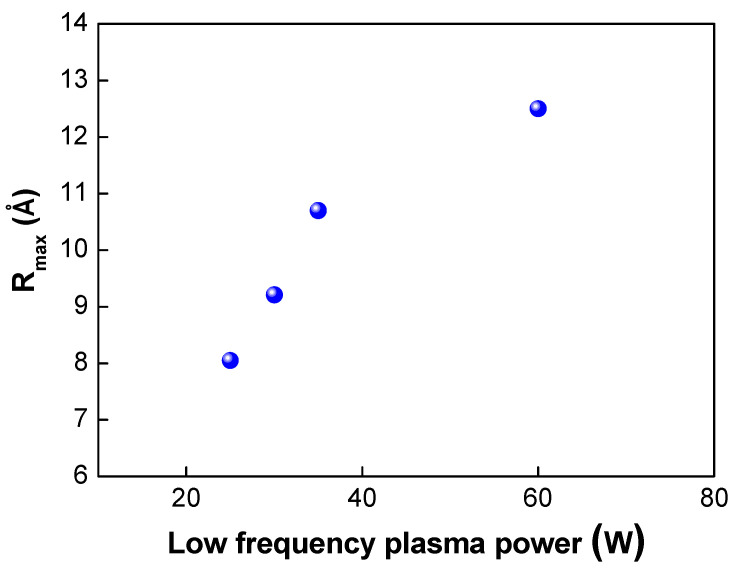
Changes in Rmax under different low plasma powers.

Next, we investigated the effects of the plasma power on the bond strength. The low-frequency plasm power was varied from 25 to 150 W. In this case, the plasma gas of oxygen was used. As shown in Table 2 and Figure 6, when the plasma power was increased from 25 to 100 W, the bond strength increased from 1.69 to 1.84 J/m^2^, As the plasma power increases, more dangling bonds form at the bonding interfaces, resulting in an increase in the bond strength. However, when the plasma power reached 150 W, the bond strength decreased slightly to 1.8 J/m^2^. Figure 7 shows the changes in the surface roughness (Rmax) with an increase in the low-frequency plasma power. As the plasma power was increased, the surface roughness increased linearly. If the plasma power exceeds a certain level, the surface roughness increases due to the ion bombardment effect in the plasma chamber, resulting in a decrease in bond strength. It is considered that there was a threshold point at which the contact area (or surface roughness) became more important for wafer bonding than the formation of dangling bonds—that is, there was a trade-off between dangling bond formation and surface roughness for plasma power. When the plasma power increased, the activity of ions also increased, resulting in more surface damage of the oxide film and an increase in the surface roughness due to the ion bombardment effects. As discussed above, an increase in the surface roughness will lead to a reduction in the real contact area and the deterioration and closing of the nanogap, resulting in a decrease in the bond strength. In addition, during the annealing process, the oxide layer will grow. If the surface roughness is significant, nanogap closing becomes less likely to occur, resulting in the generation of air trap sites between the bonded interfaces.

**Figure 8:**
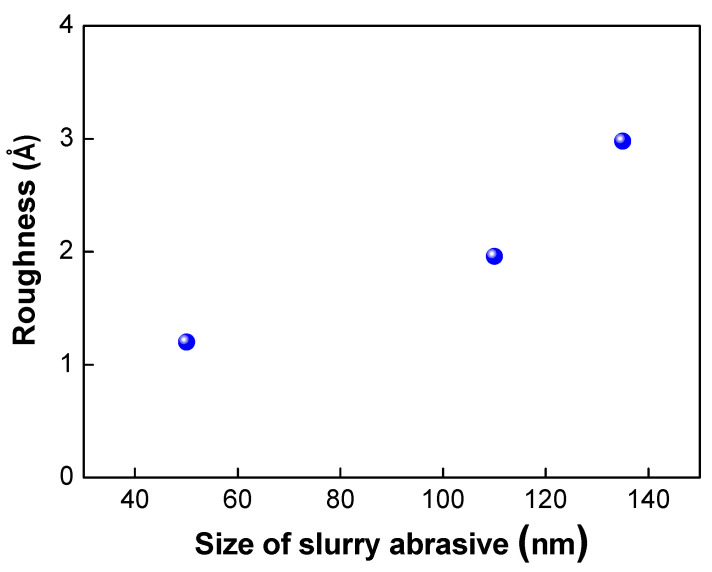
Variation of surface roughness under various slurry abrasive size.

The abrasive slurry particles used in the CMP process also play a very important role in determining the surface roughness of the wafer surface. As shown in Figure 8, as the size of the abrasive particles decreased, the surface roughness of the wafer was reduced. As discussed in the literature [21], there are many nanogaps or asperities that exist on the wafer surface. These nanogaps or asperities are one of the main sources of void formation and decrease in bond strength. Therefore, the bond strength could be improved by using smaller abrasive particles due to the greater nanogap closing effect and elimination of the asperities on the surface.

In summary, the use of O_2_ plasma gas and smaller abrasive particles will enhance the bond strength. The increase in the plasma power will also increase the bond strength by increasing the oxide growth rate. However, the much higher plasma power will instead increase the surface roughness, resulting in lower bond strength. Therefore, the proper selection of the plasma power is necessary to increase the bond strength.

**Figure 9:**
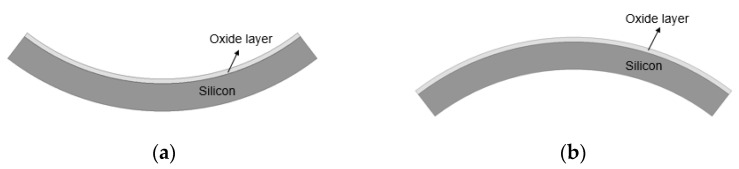
(**a**) Wafer deformed in a concave shape when the residual film stress is the tensile stress. (**b**) Wafer deformed in a convex shape when the residual film stress is compressive stress.

After the deposition process of the oxide and the copper layer, the wafer was deformed due to residual film stresses mainly caused by the mismatch of the CTE between the silicon wafer and the deposited films, which are referred to as wafer warpage. As shown in Figure 9a, the wafer will deform in a concave shape (or a smile shape) when the residual film stress is tensile stress. When the residual film stress is compressive stress, the wafer will deform in a convex shape (or a crying shape), as shown in Figure 9b.

The generation of wafer warpage is inevitable, and the warpage should be minimized, since it will cause alignment issues and the degradation of the device’s performance. However, the effects of the wafer warpage on void formation have not yet been studied.

**Figure 10:**
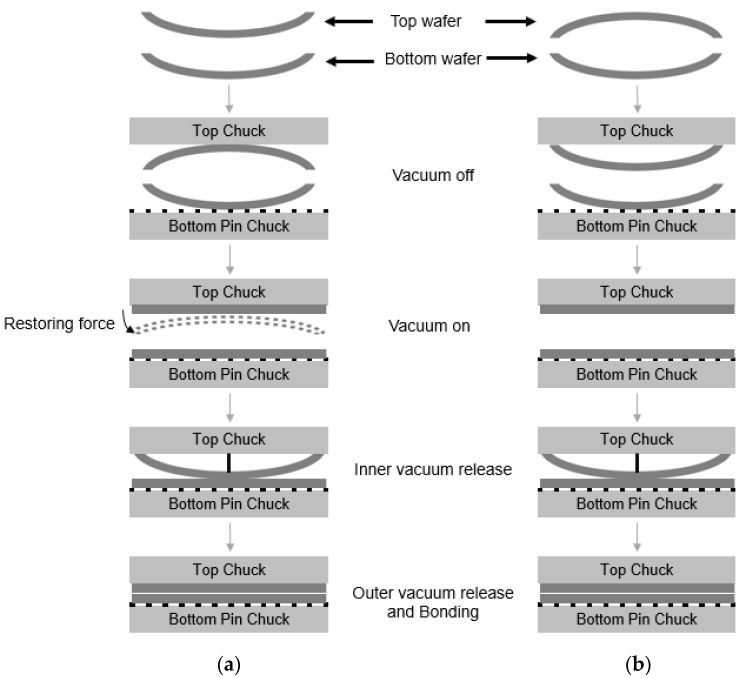
Schematic description of wafer bonding process. (**a**) Top wafer was originally deformed in a concave shape due to tensile residual film stress. (**b**) Top wafer was originally deformed in a convex shape due to compressive residual film stress.

**Figure 11:**
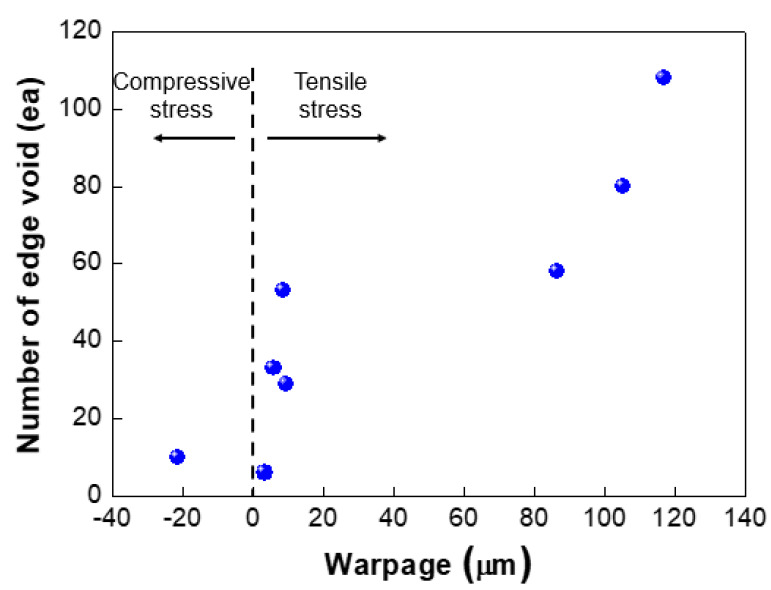
Relationship between number of edge voids and wafer warpage.

We investigated the effect of wafer warpage on the formation of edge voids. The warpage or residual film stress was controlled by changing the conditions of the deposition process. Figure 9 exhibits the wafer bonding process for wafers undergoing different residual stresses in the film. Figure 10a shows the top and bottom wafers which were deformed in a concave shape both due to tensile residual stress of the film. Figure 10b shows wafer warpage in which the top wafer was deformed in a convex shape due to compressive residual film stress while the bottom wafer was deformed in a concave shape. Figure 11 shows the relationship between the number of edge voids and the warpage of the top wafer. It was found that the top wafers with residual compressive stress (i.e., deformed in a convex shape) show fewer edge voids than those with tensile stress. With regard to positive (+) warpage with tensile stress, the number of edge voids increased as the warpage increased. This was a very interesting result, despite the fact that the physical mechanism behind it is not clearly understood at this point. However, we surmise that these results are related to the Joule–Thomson effect.

During the bonding process, the top wafer was loaded in an upside-down position into the vacuum chuck. As shown in Figure 10a, the top wafer was originally deformed in a concave shape due to the tensile residual stress of the film. During the bonding process, the top wafer with a concave shape was loaded face-down (e.g., convex shape) into the top vacuum chuck. For bonding, the vacuum was applied to both wafers, and the deformed wafers became flat. The center contact pin was then loaded onto the top wafer to come into contact with the bottom wafer, and the vacuum at the wafer edge was released. In this case, because the top wafer was deformed in a convex shape, the restoring force of the wafer edge due to the residual stress will be very high. Therefore, bonding will occur quickly. Figure 10b shows the top wafer, which was originally deformed in a convex shape. In this case, the wafer was loaded in a concave shape in the top chuck, and both the top and bottom wafer were deformed in a concave shape during the bonding process. In this case, the restoring force of the top wafer during bonding is not high; thus, the bonding speed will be slower than in the case shown in Figure 10a. As mentioned above, during the bonding process, the air between the wafer gap escapes from the center to the edge of the wafer. The air escaping the wafer gap expands at the surrounding atmospheric pressure in the wafer edge region. The rapid cooling of the air will lead to supersaturation of water, leading to the formation of a water droplet. When the bonding speed becomes high, the supersaturation of the water vapor will proceed more rapidly, increasing the possibility that a water droplet will form. Therefore, it was thought that wafer warpage is an important factor affecting the formation of edge voids.

**Figure 12:**
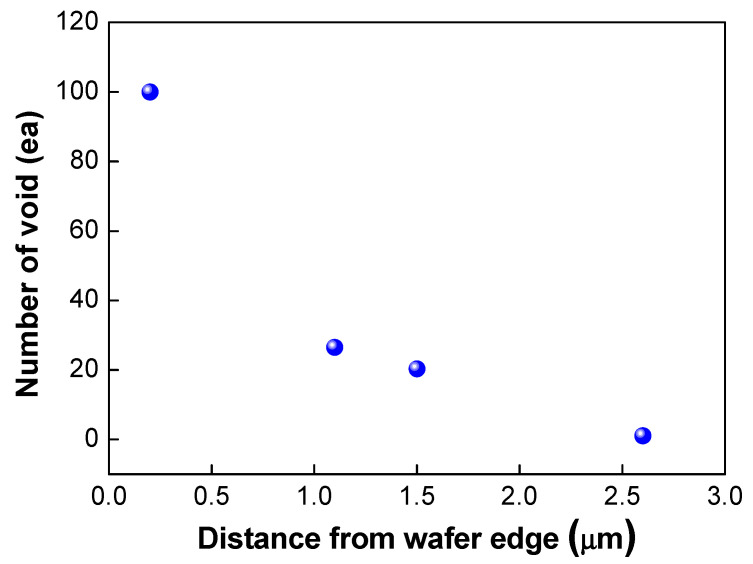
Number of edge voids and locations measured from the wafer edge.

As shown in Figure 11, edge void formation can be reduced by controlling the residual film stress of the wafer such that the convex (or crying shape) warpage of the top wafer caused by the compressive residual film stress will decrease the formation of edge voids. However, considerable wafer warpage or bow caused by residual film stress will impact the wafer-to-wafer alignment accuracy [24,25], which is the one of the critical aspects of 3D integration technology. It can be very difficult to eliminate misalignment issues completely during the bonding process, even if better alignment equipment can be developed. A room-temperature bonding process or a reduction in the annealing temperature can be beneficial to reduce wafer warpage by compensating for the CTE mismatch. We can also increase the size of the bonding pad to improve the alignment margin during the bonding process. However, this strategy is not suitable for applications involving high density levels and fine pitches. In such a case, it may be more effective to remove edge voids through a post-treatment process such as sawing or cutting. Figure 12 shows the number of the edge voids and locations measured from the wafer edge. As shown in this Figure 12, we found that edge voids were mainly located from 2.5 mm out to the wafer’s edge, which is the wafer bevel region. That is, edge voids formed due to the rapid expansion and cooling of air at the wafer edge caused by the Joule–Thomson effect, and they were located within a distance of 2.5 mm inward from the wafer edge. In general, silicon dies or chips are located 3 mm inward from the wafer edge. Therefore, even if the edge void area of 2.5 mm is removed by a sawing process, there will be no yield impact, indicating that blade sawing will be an effective method to eliminate edge voids.

## 5. Conclusions

In this study, we proposed methods to enhance the bond strength and reduce the formation of edge voids during a hybrid bonding process. The bond strength was improved when the oxide growth rate increased during the plasma process. When plasma activation was realized with O_2_ gas alone, the highest oxide growth rate and greatest bond strength were achieved. A smooth surface was also beneficial for increasing the bond strength. When the size of the abrasive particles in the CMP process was decreased, the surface roughness was reduced, leading to higher bond strength. A smooth surface leads to an increase in the real contact area and the nanogap closing, resulting in an improvement of the bond strength and a reduction in the number of voids generated. As the low-frequency plasma power was increased, the bond strength increased. However, when the plasma power was increased further, the surface roughness increased due to the ion bombardment effect during the plasma process, resulting in a reduction in the bond strength. Therefore, optimization of the plasma power will be necessary to increase the bond strength. We investigated the effect of wafer warpage on the formation of edge voids by controlling the residual stress of the oxide layer. It was found that top wafers with residual compressive stress that deformed in a convex shape exhibited fewer edge voids than those under tensile stress. These results can be explained by the Joule–Thomson effect and differences in the bonding speed. In addition, considerable warpage will increase the wafer-to-wafer misalignment during bonding, having negative effects on the yield and reliability of the hybrid bonding process. In this case, the removal of edge voids using a sawing process can be considered.

## Figures and Tables

**Table 1:** Bond strength and the thickness of the oxide layer under various plasma gas conditions.

Plasma Gas	Bond Strength (J/m^2^)	Thickness of Oxide Layer on Silicon Nitride (Å)
O_2_	1.69	33.4
N_2_	1.28	4.9
Ar	1.04	2.7
Ar/O_2_	1.36	21.3
Ar/N_2_	1.22	11.0

**Table 2:** Bond strength and thickness of the oxide layer under various plasma power conditions.

Plasma Power (W)	Thickness of Oxide Layer on Silicon Nitride (Å)	Bond Strength (J/m^2^)
25	33.4	1.69
75	43.4	1.82
100	44.4	1.84
150	64.4	1.80

## Data Availability

Not applicable.
